# The Fifteenth International Congress of Medicine

**Published:** 1904-06-04

**Authors:** 


### THE FIFTEENTH INTERNATIONAL CONGRESS OF MEDICINE.

At a recent meeting of the National Committee for Great Britain and Ireland, with Dr. Pavy, F.R.S., in the chair, Sir William Church, Bart., Sir Dyce Duckworth, Mr. Reginald Harrison, and others being present, it was resolved that, in view of the confusion and disorder attending the last assemblage of the Congress, attributable, it is considered, to the deviation from former procedure in admitting as members of the Congress others than members of the medical profession and savants, this committee deems it important, in order to avert threatened disastrous consequences, to call the attention of those entrusted with the organisation of the forthcoming Congress at Lisbon to the matter, and to urge that "no departure from the rule relating to membership in force at the Paris Congress in 1900 should be permitted to occur." This resolution was forwarded to the President and Secretary-General of the fifteenth Congress, which will be held at Lisbon in April, 1906. The rules governing the Congress have just been issued, and it is satisfactory to find that the rule as it stood at the time of the Paris Congress has been restored. The recommendations of the sub-committee as to the future constitution of the National Committee were approved, and it was decided that in future the committee should consist of a president and two secretaries who should invite the co-operation of (*a*) his Majesty's chief medical advisers in the Navy, the Army, the Indian Army, Lunacy and Local Government Boards. (*b*) The President for the time being of each medical corporation in the United Kingdom. (*c*) The President of the General Medical Council. (*d*) A professor of the faculty of medicine from each university. (*e*) The President for the time being of the Royal Medical and Chirurgical Society of London, of the Academy of Medicine in Ireland, and of the Medico-Chirurgical Society of Edinburgh. (*f*) The President for the time being of the Incorporated Society of Medical Officers of Health. (*g*) A representative of the National Medical Press Association. (*h*) Eight persons practising medicine and surgery in England, including Wales, four general practitioners practising in Scotland, and four general practitioners from Ireland. (*i*) Such other persons as it may be found desirable from time to time to invite to the National Committee. Dr. Pavy was asked to continue in the office of president, and Mr. D'Arcy Power as secretary. Dr. Horton-Smith regretted that he was unable to act as secretary to the committee. His resignation was accepted with regret and a cordial vote of thanks was tendered to him for his services. Dr. Clive Riviere was nominated in his place. It was proposed by Dr. Sutherland that, "in order to allay the feelings of irritation and disappointment rankling in the minds of those who attended the Madrid Congress and to reassure these and others who may contemplate attending the Lisbon Congress, the honorary secretaries be instructed to send a note to the medical papers to the effect that the British National Committee intend to take steps by which they hope to prevent or reduce to a minimum at the Lisbon meeting the annoyance, discomfort, and disappointment experienced in Madrid in regard to travelling facilities, lodging accommodation, etc., and to attain this end it is desirable to have a local British committee at Lisbon to keep in touch with the executive of the Lisbon Congress." The proceedings terminated with a vote of thanks to the Medical Society for its kindness in putting the Society's rooms at the disposal of the National Committee.

